# OCT Angiography to Predict Geographic Atrophy Progression using Choriocapillaris Flow Void as a Biomarker

**DOI:** 10.1167/tvst.9.7.6

**Published:** 2020-06-03

**Authors:** Khashayar Nattagh, Hao Zhou, Nicholas Rinella, Qinqin Zhang, Yining Dai, Katharina G. Foote, Cathrine Keiner, Michael Deiner, Jacque L. Duncan, Travis C. Porco, Ruikang K. Wang, Daniel M. Schwartz

**Affiliations:** 1 Department of Ophthalmology, University of California, San Francisco, CA, USA; 2 Department of Bioengineering, University of Washington, Seattle, WA, USA; 3 School of Optometry and Vision Science Graduate Group, University of California- Berkeley, Berkeley, CA, USA; 4 Francis I. Proctor Foundation, University of California, San Francisco, CA, USA

**Keywords:** OCTA, geographic atrophy, AMD, flow void, choriocapillaris

## Abstract

**Purpose:**

To investigate the relationship between choriocapillaris (CC) flow void (FV) percentage and geographic atrophy (GA) growth rate, and study how variations in FV percentage surrounding GA predict regional GA growth.

**Methods:**

This prospective, longitudinal study enrolled subjects with GA secondary to nonexudative age-related macular degeneration. Optical coherence tomography angiography imaged the CC and FV percentage was evaluated using a validated algorithm. GA growth rate was measured as the difference in the square root of GA area divided by the months between baseline and follow-up imaging.

**Results:**

Twelve eyes from 7 subjects with a mean age of 80 ± 5 years (range 74–86) were studied once at baseline and 7 to 16 months later. GA expansion rate was positively correlated with increased CC FV percentage (Spearman rank correlation coefficient *r* = 0.69 [*P* = 0.038] and 0.76 [*P* = 0.013]) within the 6 x 6 mm scanned macular region and the 2° margin surrounding each GA lesion, respectively. Regions with CC FV at baseline located within 480 µm from the GA margin showed 33% greater chance of becoming atrophic compared with regions within 480 µm from the GA margin that did not show CC FV at baseline.

**Conclusions:**

GA expansion rate and CC FV density throughout the macular region and surrounding the GA margin were significantly correlated. The regional magnitude of FV immediately surrounding GA was associated with GA growth into that region.

**Translational Relevance:**

CC FV analysis may facilitate prediction of GA growth over time for patients with advanced nonneovascular age-related macular degeneration.

## Introduction

Age-related macular degeneration (AMD) affects nearly 11 million individuals in the United States and is the primary cause of visual disability in the industrialized world; given the rapidly aging population in the United States, prevalence is expected to double in the United States by 2050.^1^ In one form of advanced AMD, geographic atrophy (GA), patients develop irreversible, progressive loss of retinal pigment epithelium (RPE), overlying photoreceptors, and choriocapillaris (CC). There are currently no approved treatments to reverse, prevent, or reduce the progression of GA.^2^ Although intraindividual progression correlates strongly with the rate of progression over the prior 2 years, and GA is strongly correlated between eyes of patients,^3^ there is high variability in GA progression among individual cases,^2^ making it challenging to predict at what rate patients will undergo progressive expansion of the GA and in what direction GA will grow, with accompanying vision loss when GA expands to involve the fovea with depletion of foveal sparing.

Although many prior studies used histology to investigate the CC in GA,^4^^–^^6^ due to recent advances in optical coherence tomography (OCT) imaging, researchers have begun searching for reliable biomarkers in the CC using noninvasive approaches.^7^^–^^13^ Historically, this has been difficult using traditional dye-based angiography techniques.^14^ The advent of OCT angiography (OCTA), however, allows for improved visualization of the CC.^15^^–^^20^ Furthermore, the development of swept-source OCTA (SS-OCTA) enables more undistorted visualization of the CC than spectral domain OCTA (SD-OCTA) by using longer wavelength light, which results in less signal attenuation by the RPE.^21^^,^^22^

Moreover, because SS-OCTA does not use a spectrometer for signal detection, the image is not susceptible to the gradual decay in sensitivity as a function of depth, which occurs with SD-OCTA.^23^ With the recent improvement of commercial SS-OCTA technology, multiple studies have demonstrated the ability to detect alterations in CC blood flow, and thus make it possible to examine new biomarkers for the prediction of GA growth.^24^^–^^28^ One such biomarker is flow void (FV) regions of nonvisible CC flow on the en face OCTA scans.^24^

By investigating regional distribution of FV in the CC, Sacconi et al.^28^ found the greatest impairment of CC vessel density surrounding the GA margin because GA tends to grow centrifugally from the margin, especially in cases of foveal sparing,^29^ in which the results suggest that CC FV density may play a role in GA progression. Supporting this hypothesis, Thulliez et al.^13^ found a significant correlation between CC FV percentage and GA growth rate. However, they reported that CC FV percentage within the entire 6 x 6 mm scan area was more strongly correlated with GA growth rate than the CC FV percentage in the regions immediately surrounding GA. These results suggest that further analysis is needed to understand the role that CC FV percentage plays in GA growth.

This study uses a quantitative analytic approach described previously to determine how FV percentage in different regions of the CC correlates with GA growth rate,^25^ and examines whether differential FV immediately surrounding GA lesions predicts regional growth of GA. We hypothesize that the regional magnitude of FV immediately surrounding GA is associated with GA growth into that region.

## Methods

### Subjects

This single-center, prospective study enrolled subjects with GA secondary to nonexudative AMD at the University of California, San Francisco (UCSF) from July 2016 to November 2018. The study was approved by the institutional review board of UCSF. Written informed consent was obtained from all subjects prior to performing any study procedures. The study was performed in accordance with the tenets of the Declaration of Helsinki and compliant with the Health Insurance Portability and Accountability Act. Inclusion criteria were the presence of GA due to AMD, clear media to permit imaging, stable central fixation, less than 6 diopters myopia, and no prior history of treatment for choroidal neovascularization. Patients with both unifocal and multifocal GA and patients with subretinal drusenoid deposits were included in this study. Exclusion criteria included macular atrophy due to hereditary retinal degenerations; retinal vascular disease; prior exposure to retinotoxic medications, including hydroxychloroquine and chloroquine; evidence of GA extending beyond the central 6 x 6 mm area scanned; and choroidal neovascularization on examination or OCT.

### Image Acquisition

The SS-OCTA system (PLEX Elite 9000; Carl Zeiss Meditec, Inc., Dublin, CA) used in this study utilized a central wavelength of 1060 nm and a speed of 100 kHz. The scan pattern used on all subjects had a field of view of 6 x 6 mm and was positioned on the center of the fovea. In patients in whom the foveal center was not clearly visible, the scan was centered on the patient's locus of fixation, which was near the fovea in all cases. This scan contained 500 A-scans x 500 locations with two repeated scans consecutively at each location,^30^ with a scanning depth of 3 mm over 1536 pixels, and transverse pixel-resolution of 12 µm. The FastTrac motion correction was enabled for all images, and the complex optical microangiography algorithm was used to generate the OCTA images.^31^^,^^32^

**Figure 1.**
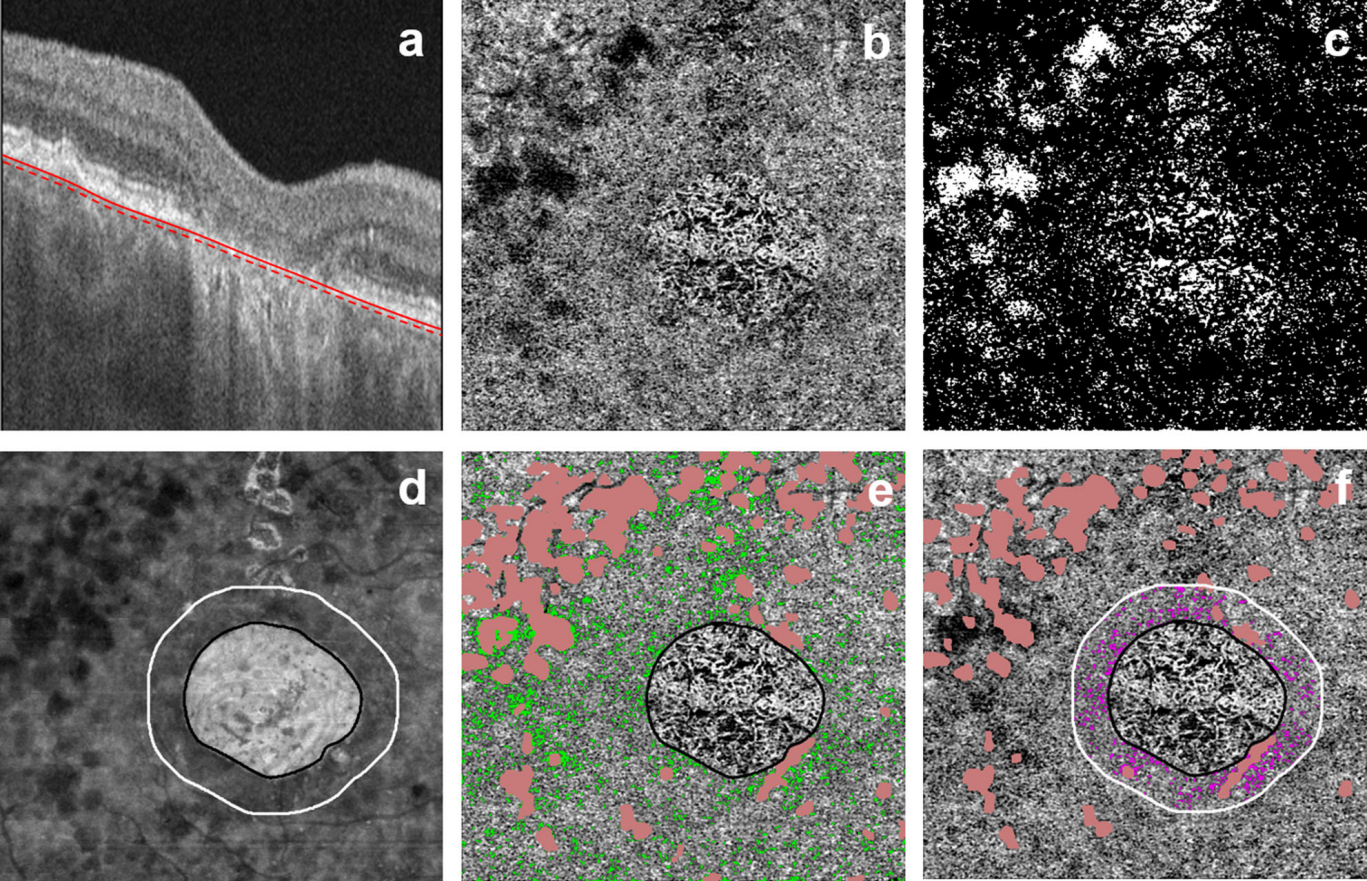
An illustration of the FV quantification process. (a) Representative OCT B-scan from subject 40118, right eye with CC segmentation; the CC was defined as a 20-µm slab beneath the outer boundary of BM (*red solid line* to *red dashed line*). (b) En face SS-OCTA 6 x 6 mm image of the CC slab. (c) Binarized CC FV map with white pixels representing segmented FVs. (d) En face SS-OCT image of sub-RPE slab with the GA outlined in black and the surrounding 2° band outlined in white. (e) En face SS-OCTA image of CC slab overlaid with ROI outside of baseline GA (*outlined in black*) with green pixels representing FV and pink pixels indicating drusen. (f) En face SS-OCTA image of CC slab overlaid with ROI between baseline GA border (*outlined in black*) and a 2° band (approximately 480 µm) outside the baseline GA (*outlined in white*). FV within the 2° margin are represented in magenta, whereas drusen are indicated in pink.

A semiautomatic segmentation algorithm developed by the coauthors^33^ was used to detect the en face CC slab (Fig. 1a), and manual corrections were made to the segmentation in cases of gross error.^33^ OCTA images were excluded if they contained significant motion or shadowing artifacts, or a signal strength less than 7 as defined by the manufacturer.

Two regions of interest in the CC were examined. One region of interest (ROI) was the entire 6 x 6 mm scan area outside the baseline GA area (Fig. 1b), and the other ROI was the area within a 2° band surrounding the baseline GA (Fig. 1c). The 2° band around the margins of GA, which is approximately 600-µm wide, was chosen to represent the area immediately adjacent to the GA margin, and we used it to compare with the remaining scanned area, excluding GA.^13^ CC FVs within these regions were evaluated by creating a threshold one standard deviation (SD) below a mean OCTA intensity determined from a normal database of 20 young subjects.^25^ FV percentage was defined in Zhang et al.^25^ as a ratio (expressed as percentage) of FV regions divided by the total ROI.

The presence of baseline GA was identified at the subject's first visit. A sub-RPE slab extending from 64 to 400 µm below the Bruch's membrane (BM) was used to generate en face structural images for GA segmentation, in which hyper-transmission of the OCT signal made the GA region appear bright in the en face image. The GA area was outlined using an automatic algorithm developed by one grader (QZ), which was then reviewed and manually corrected by another grader (HZ). B-scans were reviewed to reach consensus segmentation for all cases. The manually traced border was applied to these regions in Adobe Illustrator CC Version 2018.1.1 (Adobe Systems, San Jose, CA). Regions of GA were expressed as squared millimeters (mm^2^).

**Figure 2.**
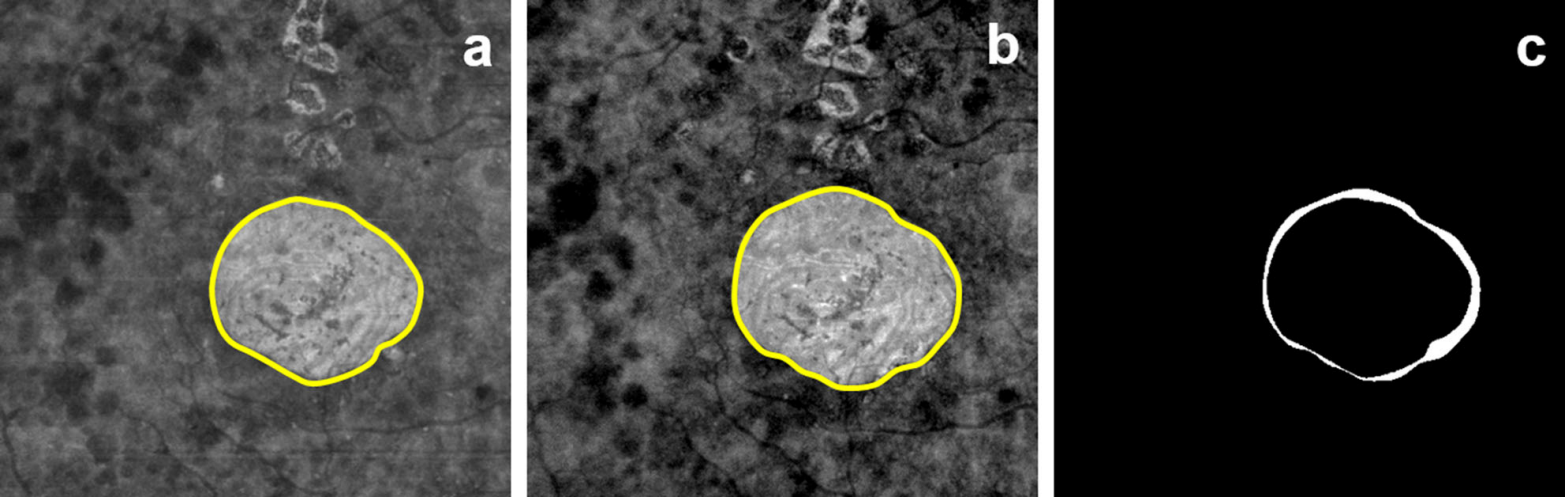
An illustration of part of the GA growth quantification process. (a) Black line outlines baseline GA from en face SS-OCT image of sub-RPE slab. (b) Black line outlines follow-up GA from en face SS-OCT image of sub-RPE slab. (c) White region indicates the difference in size of GA between baseline and follow-up GA.

The presence of GA growth was also identified (Fig. 2). The difference of the square root of areas was used to help eliminate confounding from baseline lesion size; a square root transformation used in previous studies was applied.^34^ GA growth rate was defined as the difference of the square root of area defined using OCT images between the follow-up lesion and the baseline lesion, divided by the number of months elapsed between the baseline lesion and the follow-up lesion (µm/month).

CC FV measurements were conducted using a previous method.^25^ CC was defined as a 20-µm slab beneath the outer boundary of BM and was semiautomatically segmented. Compensation for the shadowing effect caused by the RPE and BM complex on the CC slab of the OCTA was based on structural OCT information from the same slab. FVs were segmented from 6 x 6 mm en face OCTA images (500 x 500 pixels) using a threshold of 1 mean SD from a normal database of flow to define a region of FV. Regions that were excluded from analysis include areas with overlying drusen, projection artifacts from retinal vessels as previously described,^35^ and the baseline GA area as defined by the manual outline from the OCT structural image. Drusen and subretinal drusenoid deposits have been shown in previous studies to be associated with increased CC FV due to either shadowing artifacts caused by the elevated RPE/BM complex or actual CC flow reduction.^23^ Drusen were defined as localized pigment epithelial elevations on the OCT segment from the RPE to BM with a diameter larger than 25 µm^36^ to create drusen maps, which were then applied to the en face CC images. Projection artifacts from retinal vessels were removed using the en face OCTA image of inner and deep retinal slabs.^16^ CC FV in regions with projection artifacts were excluded from analysis. Similarly, FV measurements from the baseline GA were excluded by superimposing OCT images with outlined regions of GA onto subject's en face CC slab image (Fig. 1). Retinal vessels were used as landmarks on baseline and follow-up images for accurate superpositions. We assessed the relationship between FV and GA growth as follows. For each of the eyes, there were three images available: a baseline GA image derived from OCT images, a follow-up GA image outlined from OCT images, and an OCTA image depicting regions of FV at baseline. All fundus images were registered, binarized, and cropped to permit precise registration, but the central area imaged and analyzed with OCTA scans was not cropped. For the primary analysis, we examined the follow-up GA image, including all pixels from the regions bounded within 40, 60, and 80 pixels away from the boundary of the baseline GA region. The binarized images were converted to individual pixels to provide the highest possible resolution of FV surrounding the GA margins.

### Statistical Analyses

For all analyses, *P* values <0.05 were considered statistically significant. Results of quantitative analysis were expressed as mean values with SDs, 95% confidence intervals, and Spearman rank correlation coefficient. *P* values for clustered Spearman coefficients were derived by the exact permutation method, using the patient as the unit of analysis.

We used bias-reduced logistic regression to model the probability that each pixel would become part of the GA region in the follow-up image, with predictors being a second-order polynomial in distance, as well as the FV indicator at that pixel. Each eye yielded a single coefficient, summarizing the adjusted association between initial FV values and local GA status at follow-up. We tested the null hypothesis that these estimated coefficients were zero using a clustered Wilcoxon signed-rank test, accounting for the possible dependence of the two eyes within each patient. In addition to the primary analysis based on the FV image, we analyzed the results after application of Gaussian blur with a radius of 9 pixels (an arbitrary odd integer chosen to be approximately 2% of the total image size). We also conducted a model using a quadratic term in distance and a model in which we included pixels a distance of 60 pixels or closer to the baseline region. Analyses of GA expansion rates were conducted in Stata 14.1 (StataCorp, College Station, TX). All other analyses were conducted in R v. 3.4 for MacIntosh (R Foundation for Statistical Computing, Vienna, Austria; package brglm) and Julia v. 1 (http://www.julialang.org).

## Results

Twelve eyes from seven subjects with a mean age of 80 years (SD, ± 6; range, 74–86) were studied. Subjects were imaged once at baseline and again 7 to 16 months after baseline (mean, 11.5; SD, ± 2.8). Eight of 12 eyes showed multifocal GA, whereas 4 of 12 had unifocal GA lesions.

Mean FV percentage at baseline was 14.95% (SD, ± 8.10%) over the 6 x 6 mm imaged macular region (excluding areas of drusen and GA) and 18.32% (SD, ± 8.35%) within the 2° margin surrounding GA. Mean GA expansion rate was 21.37 µm/mo (SD, ± 21.13 µm/mo), corresponding to an annual rate of 256.12 µm/y (SD, ± 253.54 µm/y).

**Figure 3.**
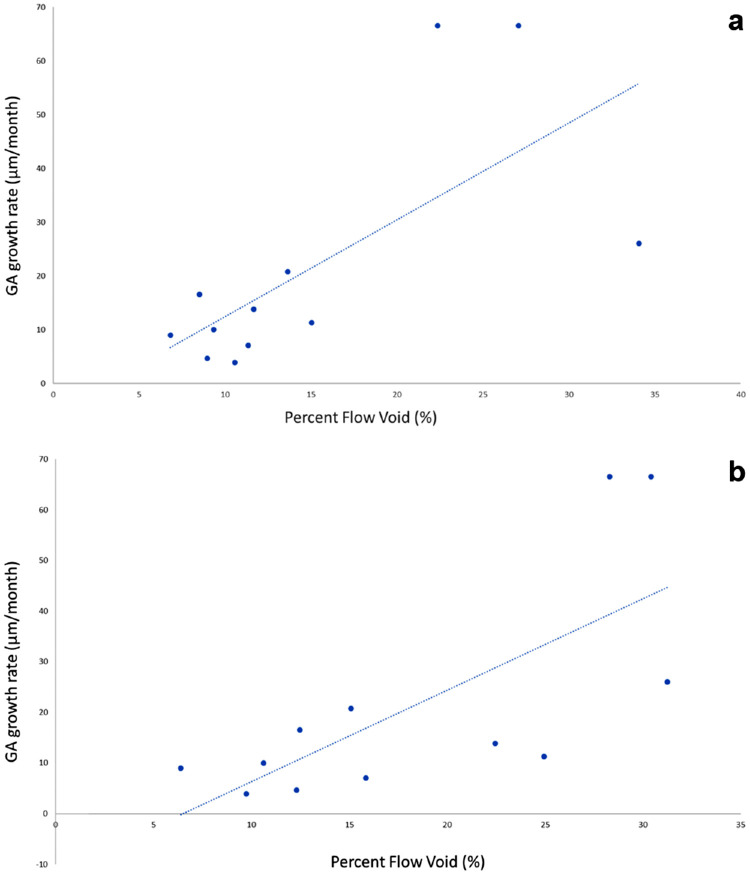
(a) Percent FV over 6 x 6 mm macular region (excluding GA lesion and regions underlying drusen) versus GA growth rate (µm/month). (b) Percent FV within 2° band outside of baseline GA margin versus GA growth rate (µm/month). Regions with drusen were excluded from analysis.

GA expansion rate was positively correlated with increased CC FV percentage (Figs. 3a, 3b). The clustered Spearman rank correlation coefficient was 0.69 (*P* = 0.038) and 0.74 (*P* = 0.013) for FV measurements within the entire 6 x 6 mm region and the 2° margin surrounding each GA lesion, respectively.

When we investigated regional GA growth as a function of CC FV percentage, we found that CC FV percentage measured to a distance of 40 pixels (approximately 480 µm) from the baseline affected region was associated with GA growth (*P* = 0.017, clustered Wilcoxon test). Specifically, 10 out of 12 eyes showed a positive adjusted coefficient for FV as a predictor of GA growth. The median of the estimated odds ratios was approximately 1.33 for pixels extending a distance of 40 pixels (approximately 480 µm) from the baseline-affected region. In other words, pixels located within a ring approximately 480 µm from the GA margin had an approximately 33% greater chance of becoming atrophic if the pixel at baseline was located in a region of CC FV compared with pixels that were not located in a region of CC FV at baseline. Similar results were seen when using the blurred flow FV predictor (*P* = 0.026, clustered Wilcoxon test). Results were unchanged when we used quadratic terms for distance from the baseline region in adjustment (*P* = 0.023), and when including regions out to 60 pixels from the baseline region (*P* = 0.017). Finally, the findings were unchanged when including three additional eyes in whom the GA lesion extended beyond the scan margins (results not shown).

## Discussion

The results of this study showed a significant positive correlation between GA expansion rate and CC FV percentage across the macular region, as well as within a 2° border surrounding each GA lesion. Correlations in both the entire macular region and region adjacent to the GA border were similar (*r* = 0.69, *r* = 0.76). For the second aim in this study, we found that the regional magnitude of FV immediately surrounding GA was associated with GA growth into that region. Specifically, we found that the chances a particular area of adjacent retina progressing to GA over approximately 1 year was approximately 33% greater if that particular area was a region of CC FV at baseline.

The GA growth rates reported in the current study are more similar to the results reported by Thulliez et al.,^13^ who found a greater correlation when measuring CC FV percentage diffusely across the macular region than with the results reported by Nassisi et al.,^7^ who found a stronger correlation when measuring CC FV percentage closer to the GA margin than more diffusely. The current study used a similar approach to that used by Thulliez et al.,^13^ who used a similar SS-OCTA system and analyzed CC FV percentage using a similar slab depth and thickness. The results differ from those reported by Nassisi et al.,^7^ who used SD-OCT to analyze a deeper slab layer, which may have resulted in greater CC FV percentage measures due to lower CC image contrast and presence of larger choroidal vessels. Although fundus autofluorescence (FAF) images are commonly used to define margins of GA, in the current study we chose to define the GA margin as regions with signal hyper-transmission at the CC layer on the OCT structural images because these were acquired simultaneously with the OCTA images. We chose this method to reduce scale and rotation error that could be introduced by aligning OCTA with FAF images, which could be magnified when comparing images longitudinally over time. Differences from prior studies could also be due to random error associated with how the GA border is outlined, or the possibility that age could be a confounding variable when measuring CC FV percentage against GA growth rate. Consistent with both prior studies, we used a square root transformation to eliminate dependency of GA growth rate on the baseline GA size.^37^

Some of the GA lesions extended beyond the area imaged with the 6 x 6 mm area included in the OCTA scan, as shown in Figure 1. The stated relationship between FVs and growth rate only pertains to those regions visible in the 6 x 6 mm standard image centered on fixation.

These data suggest that CC FV percentage, which may indicate CC hypoperfusion, is related to the rate of GA progression. Moreover, when examining regional growth of GA, we demonstrated an increased risk of GA expansion into areas with reduced CC flow at baseline. The results suggest that impaired CC FV percentage may be pathogenically important in GA progression.

The study has several limitations. The sample size of 12 eyes of 7 subjects is small, and therefore the results may not be representative of the patterns observed in other patients with AMD due to GA. Patients all had stable, central fixation and large areas of GA involving the fovea were not included in the present study. The small sample size precluded correlation with structural OCT features that have been shown to correlate with rates of GA progression, including hyperreflective foci, total drusen volume, development of internal reflectivity within drusen, and subretinal drusenoid deposits.^38^ In addition, prior studies have shown that multifocal lesions show faster rates of GA progression^39^; the number of patients in the present study was too small to make meaningful comparisons between patients with unifocal and multifocal lesions.

Future work using larger scan areas could encompass larger lesions but would sacrifice CC image resolution. In addition, patients with neovascular AMD were excluded, and the impact of choroidal neovascularization on rate of GA progression was not assessed. The present study was not able to address these and many other factors that influence the rate of GA progression, but attempted to evaluate the relationship between CC perfusion and GA growth rate with high resolution at the pixel level. Finally, when measuring FVs, each pixel corresponded to approximately 12 µm based on the manufacturer's calibration of 3.5°/mm. We acknowledge this conversion may not be accurate for all eyes, which have unique magnification parameters, but the magnification of a given eye is unlikely to change between baseline and the follow-up intervals included in the current study.

To predict GA progression, several studies have focused on measuring biomarkers found on SD-OCT images.^7^^,^^34^^,^^42^^,^^43^ Niu et al.^42^ demonstrated that thickness loss of retinal layers seen on OCT imaging may be an indicator for specific regions of GA that are more likely to progress. Nunes et al. showed that dark regions in en face SD-OCT slab images of the inner and outer segment junction may predict regions of future GA growth.^43^ Yehoshua et al.^34^ found a correlation between GA expansion rate and baseline GA area; however, when the confounding variable of lesion area was eliminated by measuring GA expansion rate as a function of the square root of the lesion area, no correlation was found. These studies illustrate the need for reliable biomarkers to predict GA growth.

OCTA permits noninvasive, longitudinal imaging of CC flow in eyes with GA, providing a glimpse of the ocular structures that perfuse the RPE and photoreceptors in living eyes. Studies of CC, BM, drusen, RPE, and photoreceptors adjacent to GA in AMD have previously been performed on histological specimens obtained postmortem, which have shown reduced CC perfusion at the margins of GA,^6^ with progressive retraction and loss of the CC with progression to GA.^5^ Histological studies have been considered the gold standard for information about anatomic structure, but can be complicated by artifact and postmortem changes, and do not permit longitudinal studies. OCTA provides in vivo, noninvasive images of CC flow, but images are complicated by shadowing from overlying vascular flow and demonstrate only vessels with blood flowing through them; vessels that persist but are nonperfused or slowly perfused are not reliably visualized.

## Conclusions

Using these techniques to produce a real-time, noninvasive prediction for GA growth could be useful clinically. Analysis of regions of CC FV around GA margins may allow for the estimation of speed and the direction of GA growth. This could be especially useful for patients with foveal sparing and parafoveal GA lesions concerned with future central visual loss.^46^ Thus future work could automate FV quantification, allowing for clinically useful, real-time estimation of GA growth rate and regional growth patterns. If CC hypoperfusion is in fact contributing to GA progression, then efforts should be made therapeutically to enhance CC perfusion and potentially slow progression of GA.
